# Engineered sequestrins inhibit aggregation of pathogenic alpha-synuclein mutants

**DOI:** 10.3389/fimmu.2025.1574755

**Published:** 2025-05-16

**Authors:** Linnea Charlotta Hjelm, Wojciech Paslawski, Christofer Lendel, Siri Flemming Svedmark, Per Svenningsson, Stefan Ståhl, Hanna Lindberg, John Löfblom

**Affiliations:** ^1^ Department of Protein Science, School of Engineering Sciences in Chemistry, Biotechnology and Health, KTH Royal Institute of Technology, Stockholm, Sweden; ^2^ Department of Clinical Neuroscience, Karolinska Institute, Stockholm, Sweden; ^3^ Department of Chemistry, School of Engineering Sciences in Chemistry, Biotechnology and Health, KTH Royal Institute of Technology, Stockholm, Sweden

**Keywords:** affibody molecule, alpha-synuclein, directed evolution, Parkinson’s disease, phage display, sequestrin

## Abstract

Misfolding and aggregation of the neuronal protein alpha-synuclein (aSyn) has been identified as a hallmark of Parkinson’s disease (PD) pathology and other synucleinopathies. Preventing formation of intracellular aSyn accumulations constitutes a therapeutic strategy against disease development. We recently reported on a new type of affinity protein, denoted *Sequestrin*, aimed for efficient and stable interactions with aggregation-prone amyloidogenic proteins and peptides. Upon binding, sequestrins interact with the aggregation-prone peptide and form a stabilizing four-stranded beta sheet with similarities to the beta sheet rich structures seen in amyloid fibrils. Here, high-affinity aSyn-binding sequestrins were isolated from a large naïve sequestrin library using phage display technology. The best binders demonstrated dissociation constant, K_D,_ values in the 10 nM-range, and structural rearrangements in both the sequestrin and aSyn protein upon binding. Modelling using AlphaFold, followed by NMR spectroscopy suggested that the sequestrins bind an N-terminal region of aSyn that is critical for amyloidogenic aggregation. In an *in vitro* aggregation study, the sequestrins demonstrated complete inhibition of aSyn aggregation at equimolar concentrations, including the three familial mutants A30P, E46K, and A53T that are associated with Parkinson’s disease and Lewy body dementia.

## 1 Introduction

Neurodegenerative disorders (NDDs) are a family of diseases of which neurons of the brain are progressively degraded, resulting in various clinical symptoms, such as impaired motor and cognitive functions. Parkinson’s disease (PD) is the second most common NDD, affecting over 1.2 million people (1–3). The exact cause of the disease remains unknown, however, the main neuropathological hallmark is the presence of cytoplasmic Lewy bodies (LB). A major component of LBs is misfolded and aggregated forms of the pre-synaptic protein alpha-synuclein (aSyn) (1, 4). While alpha-synuclein is the most extensively studied member of the synuclein family due to its strong association with Parkinson’s disease, the physiological and pathological roles of the homologous proteins’ beta-synuclein and gamma-synuclein remain less well defined. Emerging evidence suggests that these proteins may modulate aSyn aggregation or contribute to distinct neurodegenerative processes (6).

Alpha-synuclein is a 140 amino acid (14 kDa) presynaptic protein involved in synaptic plasticity, vesicular handling, and neurotransmitter release. The protein comprises three distinct regions that include an N-terminal lipid-binding region, the central non-amyloid-β component (NAC) segment, and an acidic C-terminal region. In its soluble form, aSyn is primarily monomeric and disordered, and localizes at the presynaptic terminals. Misfolding and aggregation of aSyn result in formation of β-sheet-rich oligomeric and fibrillar structures found throughout neuronal cells (5). Aggregation of aSyn is polymorphic and driven by either the β-hairpin region (amino acid 36–57) or the hydrophobic NAC domain (amino acid 61–91) (7, 8). Both types of aggregates induce synaptotoxicity by impairing synaptic plasticity and causing pores in the cell membrane, ultimately leading to cellular death. This neuronal loss results in reduced dopamine levels, which drives the on-set of clinical symptoms (1, 9). Toxicity of aSyn in neurodegeneration is highlighted by increased expression or the presence of N-terminal mutations (A30P, E46K, H50Q, G51D, A53E and A53T) in the aSyn encoding gene, resulting in higher aggregation propensity (5). Moreover, aSyn has been demonstrated to spread between neuronal cells in a prion-like manner. Consequently, preventing aSyn spreading and aggregation, or reducing the extent of aSyn pathology, represent interesting neuroprotective strategies for therapeutic intervention (10).To date, available drugs for PD only slow the progression of the disease. The clinical development pipeline for PD is diverse, encompassing various targets, mechanisms, and drug delivery systems. In recent years, a leading focus has been on addressing aSyn pathology (11, 12). Given the complexity of the disease, antibodies targeting different conformations of extracellular aSyn are in development for therapeutic and diagnostic purposes. Examples include MEDI1341 (13) targeting monomers and aggregated forms; Cinpanemab (14) binding oligomers; ABBV-0805 (15) binding oligomers/protofibrils; and Lu AF82422 (16) binding fibrils. Two other prominent disease-modifying candidates in development are the monoclonal antibody Prasinezumab that targets soluble and aggregated aSyn (17–19), and the neuroprotective peptide Liraglutide (Victoza, repurposed from diabetes treatment) (20). Encouraging findings from a recent study suggest that Prasinezumab slow the progression of motor symptoms to some extent (21). Antibody derivatives and engineered small protein scaffolds have been developed as interesting alternatives to monoclonal antibodies. Such alternatives are generally associated with a much smaller molecular size that allows more straight-forward engineering of various traits, for example multispecificity (22, 23).

Affibody molecules are 6.5 kDa alternative scaffold proteins with a three-helical bundle structure (23). New affibody molecules are typically generated through directed evolution [e.g. bacterial or phage display (24–26)] and binders to more than 60 different targets are reported in literature (23). Currently, the most advanced therapeutic affibody construct, izokibep, targeting interleukin-17A is in late clinical trials for several indications (www.affibody.se), demonstrating excellent safety profiles and efficacy (27). For therapeutic applications, such as with izokibep, an albumin-binding domain (ABD) is commonly fused to the affibody to prolong the half-life in blood circulation (28, 29). Efforts have also focused on generating affibody molecules targeting intrinsically disordered peptides, including the Alzheimer’s-related amyloid beta (Aβ) peptide. Interestingly, these selections resulted in an atypical class of disulfide-linked dimeric affibody binders with a novel target-binding mechanism. Upon binding, the two affibody domains interact with the aggregation-prone Aβ peptide and form a stable four-stranded β-sheet structure together with the peptide, which adopts a β-hairpin conformation similar to that found in amyloid fibrils. The complex is further stabilized by complete sequestering of the aggregation-prone residues of the peptide in a tunnel-like hydrophobic cavity that is formed between the two affibody domains (30–32). Recently, an engineered high-affinity Aβ-binding variant (K_D_ ~60 pM), denoted Z_SYM73_, was evaluated for preventive treatment in an APP/PS1 transgenic mouse model of AD. The study demonstrated rescued cognitive functions as well as prevention of amyloid plaque burden in cortex and hippocampus of animals treated with the construct. Importantly, no toxicological symptoms or immunological side-effects were observed (33, 34).

Based on these encouraging results, we recently designed a new type of affinity protein, denoted *Sequestrin (Sq)*, aimed for efficient interaction with aggregation-prone amyloidogenic proteins and peptides. The scaffold was engineered into a head-to-tail genetic dimer that was truncated in the N-terminus. Based on sequence and structure analysis of previous dimeric affibody-based molecules in complex with aggregation prone peptides, eleven positions in each subunit were selected for randomization to construct a new combinatorial library. In a proof-of-concept study, the quality of the library scaffold and design was assessed by generating new high-affinity candidates against soluble, monomeric Aβ using phage display technology (35).

**Figure 1:**
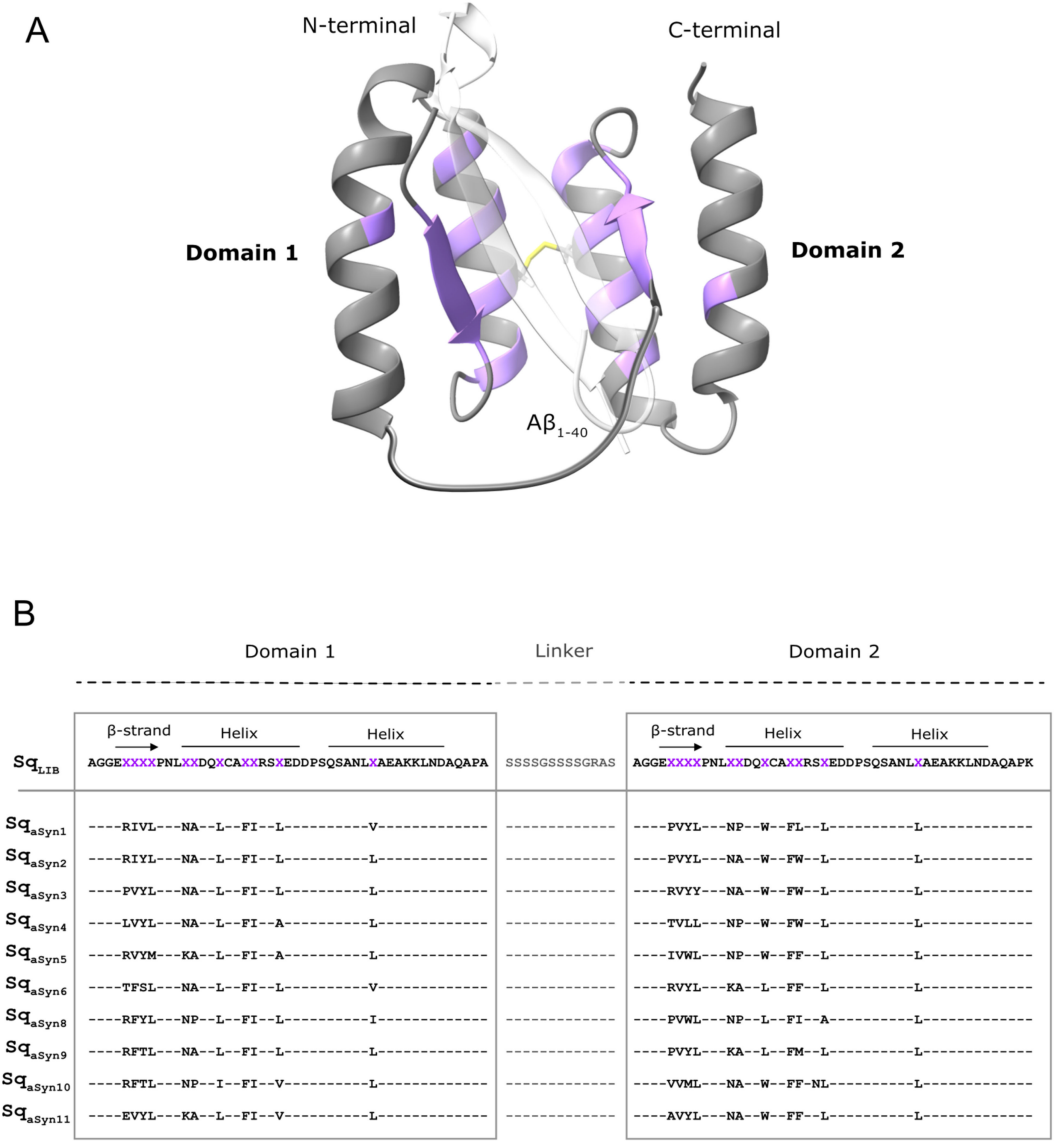
Schematic overview of the sequestrin library and phage-display selected sequences against aSyn_1-140_. **(A)** Illustration of the sequestrin (Sq) library with the two domains genetically connected by a flexible linker (grey), a disulphide bridge (yellow) and highlighting the eleven randomized positions in each domain (purple). The structure is based on PDB entry 2OKT and using the Alzheimer´s-associated amyloid beta (Aβ_1-40_) peptide (white-transparent) as a model peptide. **(B)** The amino acid sequence of the sequestrin library (Sq_LIB_) and ten phage-display selected candidates towards aSyn. Randomized residues are shown as “X” (purple).

Here, the new sequestrin library (**Figure 1A**) was used to generate high-affinity aSyn-binding candidates, aimed to sequester the aggregation-prone regions of aSyn. Characterization of candidates from the selection demonstrated affinities in the low nanomolar range. Spectroscopic analysis of secondary structure content suggested structural rearrangements in both the sequestrins and aSyn upon binding, consistent with previous findings on similar types of binders (32, 35). AlphaFold modelling predicted a β-hairpin conformation of aSyn in the sequestrin complex, and NMR spectroscopy confirmed that the sequestrins interact with the N-terminal region of aSyn, which is a critical region for aggregation. Notably, the sequestrins demonstrated complete inhibition of aSyn *in vitro* aggregation at equimolar concentrations, including the three familial PD mutants A30P, E46K, and A53T.

## 2 Materials and methods

### 2.1 Phage display selections of sequestrins binding to alpha-synuclein

Phage display selections of sequestrins binding to alpha-synuclein_1-140_ (aSyn_1-140_) were performed from a large combinatorial phage library, essentially as described previously (35). Briefly, five rounds of bio-panning were performed using biotinylated aSyn (ALN-H82H8, ACRO Biosciences, Newark, DE, USA). Each round was conducted at 4°C in PBSTB (PBS with 0.05% Tween20 and 3% w/v BSA) with decreasing target concentrations (200 nM, 200 nM, 100 nM, 50 nM and 25 nM) and increased washing. In the first round, the library was incubated with target overnight, and in rounds 2–5 for 1 hour followed by binding onto paramagnetic streptavidin beads (M-280, Thermo Fisher Scientific). In the last round, the library was divided on two parallel tracks with one incubated at 4°C and the other at room temperature. Target-bound phages were eluted using glycine-HCl (pH 3.0, 0.3 M), followed by neutralization with Tris-HCl (pH 8.0, 1 M). Target binding (of phage pools after each selection round, or 99 individual clones after the last selection rounds) was tested in a polyclonal or monoclonal ELISA format, respectively. ELISA screening was performed as previously described (35) and with 5 μg/ml streptavidin (SA, Thermo Fischer Scientific, Waltham, USA) immobilized for subsequent incubation with 5 μg/mL biotinylated aSyn. Separate wells were also immobilized with bovine serum albumin (BSA, 1 w/v%) as negative control, or 5 μg/ml human serum albumin (HSA, Sigma Aldrich/Merck, Solna, Sweden) for assessment of proper display of the expression cassette containing a genetic fusion of the sequestrin library to an albumin-binding domain (ABD) and truncated protein 3. Binding signals were normalized to the HSA signal and blanked by subtraction from BSA or streptavidin–BSA background signal. DNA sequences were identified with Sanger sequencing (Microsynth SeqLab, Göttingen, Germany).

### 2.2 Production, purification and characterization of recombinant sequestrins

Gene fragments encoding ten sequestrins (Sq) were PCR amplified from phagemids for cloning into the expression vector pET-26b(+) (Novagen, San Diego, CA) for periplasmic translocation, and introducing a C-terminal hexahistidine tag for immobilized metal affinity chromatography. Constructs were of the format Sq-His_6_. Constructs of the format Sq-ABD_035_-His_6_ were prepared by cloning into a modified version of the pET26b(+) containing a C-terminal albumin-binding domain (ABD_035_) followed by hexahistidines (28). Sequence-verified clones (Sanger sequencing, Eurofins Genomics, Ebersberg, Germany) were transformed by heat shock to the *Escherichia coli* strain BL21 star (Thermo Fisher Scientific Waltham, MA, USA), followed by protein expression and purification. Briefly, cells were cultivated in tryptic soy broth with yeast extract (TSB+Y; Merck) supplemented with 25 μg/ml kanamycin and grown at 37°C with 150 rpm shaking. At an OD_600_ of approximately 0.7, protein expression was induced with 1 mM isopropyl β-D-1-thiogalactopyranoside (IPTG; Thermo Fisher Scientific). Cultures were incubated at 25°C with 150 rpm shaking for 16 hours prior to harvest. Cells were resuspended in 50 mM NaP buffer [47 mM Na_2_HPO_4_, 3 mM NaH_2_PO_4_, 300 mM NaCl, 15 mM imidazole, pH 7.4], lysed by sonication, and harvested by centrifugation. The lysate was filtered (0.45 μm), added to HisPur Cobalt Resin (Thermo Scientific, Waltham, MA, USA), and His_6_-tagged sequestrins were eluted with 150 mM imidazole followed by buffer exchange to PBS (pH 7.4) on PD-10 desalting columns (Cytiva, Marlborough, MA, USA), according to manufacturer’s recommendations. Protein concentrations were measured using a Pierce BCA Protein Assay Kit (Thermo Scientific, Waltam, MA, USA), according to manufacturer’s instructions. 1.5 μg of each protein was analyzed on a Sodium Dodecyl Sulphate Polyacrylamide Gel (SDS-PAGE, NuPAGE Bis-Tris 4–12%, Invitrogen, Waltham, MA, USA) at oxidizing conditions. Molecular masses were determined by mass spectrometry (MS, Thermo Ultimate3000 Bruker Impact II, Thermo Fisher).

### 2.3 Biosensor-based screening and ranking of aSyn_1-140-_binding sequestrins

Purified sequestrins (Sq-His_6_) were screened for binding to aSyn_1–140_ by surface plasmon resonance (SPR) on a Biacore 8K system (Cytiva, Marlborough, MA, USA) and using PBST as running buffer (0.05% Tween20). Biotinylated aSyn_1–140_ (ALN-H82H8, ACRO Biosciences, Newark, DE, USA) was immobilized to 190 RU on a Series S SA sensor chip (Cytiva). Each Sq-His_6_ was injected in four concentrations (3000, 1500, 750, and 375 nM) for 350 seconds at 30 μl/min and 25°C or 37°C. Dissociation was monitored for 1,400–1,500 seconds, before regeneration with 10 mM Glycine-HCl pH 2.4 (Sigma-Aldrich, St. Louis, MO, USA) for 35 seconds at 30 μl/min. Samples were run in duplicates, and the kinetic constants were estimated using a Multi-cycle kinetics method with 1:1 binding and 1:1 dissociation using the Biacore Insight Evaluation Software (Version 2.0.15, Cytiva, Marlborough, MA, USA).

### 2.4 Circular dichroism spectroscopy for secondary structure and melting temperature determination of Sq_aSyn_:aSyn complexes

Secondary structure content of free sequestrins (Sq-His_6_ format) or aSyn_1-140_ (ALN-H82H8, ACRO), was analyzed using circular dichroism (CD) spectroscopy on a Chirascan Circular Dichroism Spectrometer (Applied Photophysics Ldt, Leatherhead, United Kingdom). Analysis was performed using a protein concentration of 0.2 mg/ml of the sequestrins or 15.7 μM of aSyn_1–140_ in PBS (pH 7.4) and with a 1 mm High precision cell (110-1P-40 cuvettes, Hellma Analytics, Germany). Secondary structure content was assessed by measuring ellipticity between 195 nm and 260 nm at 20°C. Melting curves were recorded by measuring the change in ellipticity at 221 nm while heating from 20°C to 90°C and using a temperature gradient of 1°C per minute. After cooling to 20°C, refolding capacity was assessed by recording another spectrum.

Secondary structure analysis before and after heat-induced denaturation of Sq_aSyn_:aSyn complexes were performed by co-incubation of equimolar concentrations (15.7 μM) of the proteins in PBS (pH 7.4), followed by assessment as described above.

### 2.5 Production of recombinant soluble aSyn monomeric proteins

Recombinant human wild type (wt) aSyn_1–140_ and familial variants A30P, E46K, and A53T were expressed in *E. coli* and purified as described previously (36, 37). Briefly, the expression vector pET11-D, containing the insert coding for human aSyn was expressed in *E. coli* BL21 (DE3) competent cells using an auto-induction method. Cells were harvested by centrifugation and incubated with osmotic shock buffer (20 mM Tris-HCl, pH 7.2, 40% sucrose) for 10 min, followed by centrifugation. The cell pellet was suspended in ice-cold deionized water, followed by addition of saturated MgCl_2_, and briefly incubated on ice. The periplasmic fraction of the cell lysate was collected, and the majority of incorrect proteins were precipitated by acidification. The solution was fractionated on a Q-Sepharose column connected to an ÄKTA Explorer system (Cytiva, Marlborough, MA, USA). Fractions containing aSyn were identified by SDS-PAGE, pulled together and high molecular weight aggregates were removed by filtration through a 30 kDa filter. The aSyn concentration was determined using NanoDrop ND1000 (Thermo Fisher Scientific, Waltham, MA, USA), and the protein was aliquoted, lyophilized and stored at −20°C.

### 2.6 Biosensor-based analysis of sequestrins binding to aSyn mutants

Sequestrins in the format Sq-ABD_035_-His_6_ were analyzed for binding to wt aSyn_1–140_ and three disease-related mutants A53T, A30P, or E46K on a T200 instrument (Cytiva) and using PBST as running buffer. A CM5 chip (Cytiva) was immobilized with 2000 RU human serum albumin (HSA, Thermo Fisher) using amine coupling, and 10 mM sodium acetate pH 4.5 as immobilization buffer. 50 RU of each Sq-ABD_035_-His_6_ was captured on the HSA surface for 60 sec at a flow of 10 μl/min before injection of each of the aSyn protein variants wt, A53T, A30P, or E46K at five different concentrations (24,000, 8000, 2667, 888, 296 nM) and in single cycle dilution series for 250 sec at 30 μl/min flow. Dissociation was monitored for 1800 sec prior to regeneration with 10 mM Glycine-HCl pH 2.4 (Sigma-Aldrich) for 70 sec at 30 μl/min with a 60 sec stabilization time.

### 2.7 Nuclear magnetic resonance of Sq_aSyn4_:aSyn complex

Uniformly ^15^N-labeled aSyn was produced as previously described (38). Nuclear magnetic resonance (NMR) samples were prepared in 10 mM sodium phosphate buffer with 50 mM NaCl, pH 7.4 and contained 5-10% ^2^H_2_O. Data were recorded on a Bruker Avance 700 MHz spectrometers equipped with cryoprobe, processed using TopSpin (Bruker) and analyzed in CCPNMR (39). The experiments were performed at 10°C.

2D ^1^H-^15^N heteronuclear single quantum correlation (HSQC) (40) spectra were recorded with 1748 x 128 complex points and spectral widths of 10 and 26 ppm in the ^1^H and ^15^N dimensions respectively. The HSQC spectrum of 130 μM ^15^N-aSyn was monitored during addition of 0.17, 0.33, 0.67 and 1.0 molar equivalents of Sq_aSyn4_. Spectral assignment of the ^1^H-^15^N HSQC of free aSyn was based on previous work (41–43). The assignments of the spectra in the presence of Sq_aSyn4_ were obtained by following the peaks in the correlation map during the titration.

### 2.8 In silico protein structure predictions

Protein structure predictions were generated using AlphaFold3 (44) and visualized in ChimeraX software (45).

### 2.9 Thioflavin T aggregation assay

Thioflavin T fluorescence of aggregating aSyn, including wt and familial variants A30P, E46K, and A53T, was recorded in 96-well plates (Nunc) and using a FLUOstar Omega plate reader (BMG Labtech, Ortenberg, Germany). Wt aSyn_1–140_ or the three mutants A30P, E46K, and A53T were individually incubated at 70 μM with 40 μM Thioflavin T dye (ThT, AnaSpec, San Jose, CA, USA), with or without the addition of the respective sequestrins (Sq_aSyn2_, Sq_aSyn3,_ Sq_aSyn4,_ Sq_aSyn11_) at concentrations of 70, 14 or 7 μM. Prior to co-incubation, sequestrins were additionally purified by size exclusion chromatography (SEC) using an ÄKTA system and a HiLoad 16/600–200 pg column (Cytiva) with PBS as running buffer. Plates were sealed with polyolefin tape (Nunc) and incubated at 37°C. Data points were measured during 72 h with orbital shaking before measurements at 448 nm excitation and 482 nm emission.

## 3 Results

### 3.1 Isolation of alpha-synuclein-binding sequestrins by phage display

In order to generate aSyn-binding sequestrins, phage display selections were performed from a large combinatorial library, in principle as previously described (35). Briefly, an M13 filamentous phage library of 5×10^9^ candidates and with 22 partially randomized positions (**Figures 1A, B**) was subjected to five rounds of bio-panning against biotinylated aSyn_1-140_. In each cycle, selection stringency was increased by decreasing concentration of target (200 nM, 200 nM, 100 nM, 50 nM and 25 nM) and by increasing number of washes. The first four cycles were carried out at 4°C to minimize aggregation of aSyn during the selection. In the fifth cycle, the phage pool was divided in two tracks for panning both at 4°C and room temperature. DNA sequencing of positive phage-ELISA clones from the last cycles of selection demonstrated enrichment of mainly heterodimeric candidates. Interestingly, a trend of non-polar amino acids in the β-strand motif of domain 1 was observed, while more hydrophobic amino acids were introduced in the second domain (**Figure 1B**). Moreover, a few positions amongst the selected sequestrins appeared conserved in all selected candidates (**Figure 1B**).

### 3.2 Production, purification and characterization of recombinant sequestrins

Ten candidates, representing the major sequence clusters from the phage display selection against aSyn (**Figure 1B**) were produced with a C-terminal His_6_-tag. Briefly, the gene sequences encoding the constructs were subcloned into an expression vector and produced in *E. coli*. Cells were lysed by sonication and purified to homogeneity by immobilized metal affinity chromatography (IMAC). The molecular weights and purity of the proteins were confirmed by SDS-PAGE (**Supplementary Figure S1**) and mass spectrometry. Secondary structure content, thermal stability and refolding capability after denaturation were assessed by circular dichroism (CD) spectroscopy. The analysis revealed that all candidates predominantly adopt an alpha-helical secondary structure (**Supplementary Figure S2A**) and exhibit melting temperatures ranging from 38 to 48°C (**Supplementary Figure S2B**). Additionally, all sequestrins demonstrated the ability to refold after heat-treatment (**Supplementary Figure S2A**).

### 3.3 Biosensor-based screening and ranking of sequestrins binding to aSyn_1-140_

**Figure 2:**
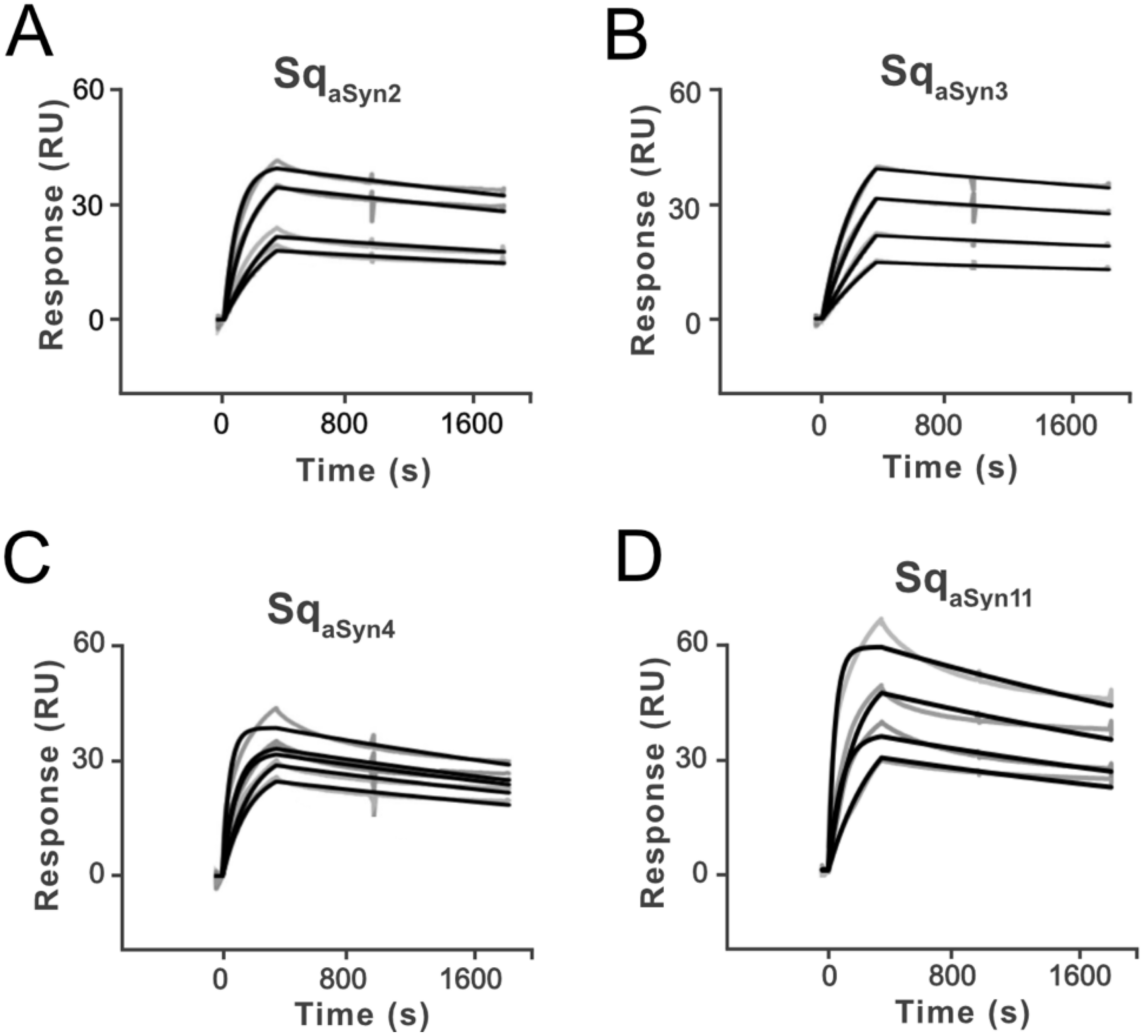
Surface plasmon resonance (SPR) sensorgrams showing the interaction of aSyn_1-140_, captured on a sensor chip, and four sequestrins Sq_aSyn2_ **(A)**, Sq_aSyn3_ **(B)**, Sq_aSyn4_ **(C)** and Sq_aSyn11_ **(D)**, respectively, at 37°C. Grey curves are referenced raw data and black lines are fitted curves. All analytes were run in duplicate at concentrations from 3000 nM to 375 nM in dilution series. Equilibrium dissociation constants for the interaction between each sequestrin and aSyn were estimated using a 1.1 Langmuir model fit.

Surface plasmon resonance (SPR) was used to rank the ten sequestrins based on aSyn_1–140_ binding at 25°C. Briefly, C-terminally biotinylated aSyn was captured on a streptavidin-coated sensor chip, followed by injection of each of the ten sequestrins, respectively. A trend of both slow association and slow dissociation was observed across all candidates (**Supplementary Figure S3**). The slow association is likely attributed to structural rearrangements in both the sequestrins and aSyn protein upon complex formation, as has been previously reported for other sequestrins (35). Four candidates, Sq_aSyn2_, Sq_aSyn3_, Sq_aSyn4_, and Sq_aSyn11_, were selected for further analysis at 37°C (**Figures 2A–D**). As expected, the kinetics of the sequestrins binding to aSyn was faster at this temperature, reflecting increased molecular dynamics. Nevertheless, the affinities remained consistent with observations at 25°C and the K_D_ values for these four candidates ranged from approximately 12 to 30 nM (**Supplementary Table S1**).

### 3.4 Circular dichroism spectroscopy for secondary structure and melting temperature determination of Sq_aSyn_:aSyn complexes

**Figure 3:**
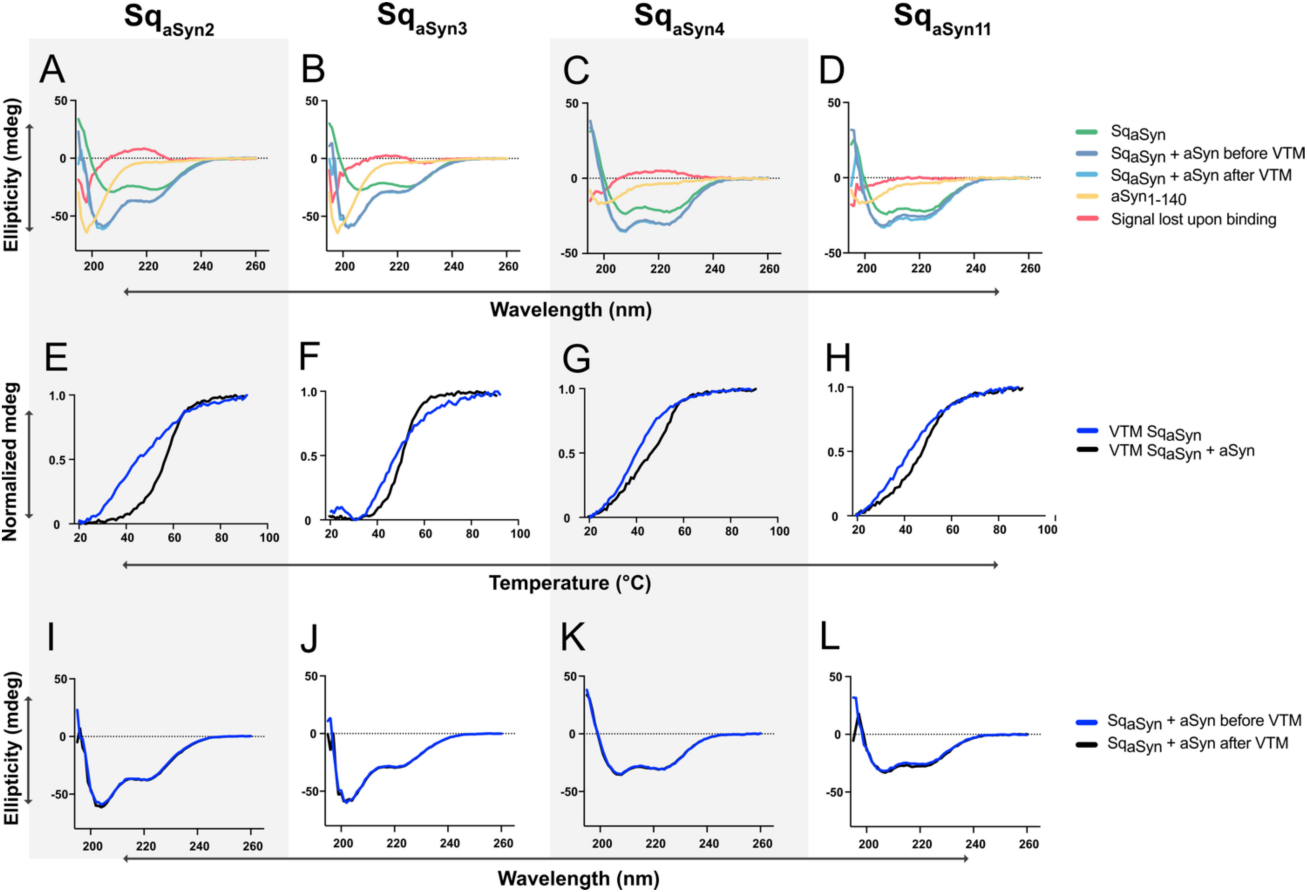
Circular dichroism (CD) spectroscopy characterization, including secondary structure components and variable temperature measurements (VTM), of four Sq_aSyn_:aSyn_1–140_ complexes. **(A-D)** Secondary structure content of free aSyn_1-140_ (yellow), free sequestrins (green), Sq_aSyn_:aSyn complexes at equimolar concentrations (blue), and the structure that was lost upon binding (red), i.e. the calculated difference between the sum of the free spectra and the complex spectrum. **(E-H)** Variable temperature measurements (VTM) between 20-90°C at 221 nm for the sequestrins (blue) or in complex with 15.7 μM molar equivalents of aSyn (black). **(I-L)** CD spectra for sequestrins co-incubated with molar equivalents of aSyn before (blue) and after (black) heat-induced denaturation.

Sequestrins have previously been shown to undergo structural rearrangements upon binding to their targets (32, 35). To investigate whether similar changes occur upon interaction with aSyn, circular dichroism (CD) spectroscopy was used to analyze secondary structure content of the four sequestrins Sq_aSyn2_, Sq_aSyn3_, Sq_aSyn4_ and Sq_aSyn11_, both in their free form and when co-incubated with aSyn. The secondary structure of free aSyn was first assessed, showing a characteristic random coil conformation, which remained unchanged both before and after heating to 95°C (**Supplementary Figures S4A, B**). The four sequestrins demonstrated predominantly alpha-helical secondary structure, in line with previously reported structural characteristics of sequestrins (**Figures 3A–D**, green). Next, CD analysis was performed on samples containing aSyn co-incubated with each of the four sequestrins. By subtracting the sum of the ellipticities of the free proteins from the signal of the complexes, residual spectra with minimum at 201 nm were observed (**Figures 3A–D**, red). This residual signal is indicative of a random coil structure and reflects the structural elements that were lost during complex formation. Interestingly, the complexes involving Sq_aSyn2_ and Sq_aSyn3_ showed an increase in ellipticity around 215 nm, indicative of a gain in β-sheet structure and suggesting the formation of additional β-sheet elements upon binding. The thermal stability of the complexes was also assessed using variable temperature measurements (VTM). The melting temperatures (T_m_) of all sequestrin:aSyn complexes were slightly higher than those of the free sequestrins (**Figures 3E–H**), indicating that complex formation may have a stabilizing effect on the proteins. Additionally, heat-induced denaturation experiments demonstrated that the complexes retained the ability to refold after heating (**Figures 3I–L**).

### 3.5 Surface plasmon resonance for analysis of dissociation of sequestrins from aggregation-prone alpha-synuclein mutants

To investigate the potential of the sequestrins to bind three familial aSyn mutants (E46K, A30P and A53T), a biosensor-based setup was employed. Monomeric recombinant human aSyn_1-140_ (wild type and familial variants) was prepared using ion exchange chromatography and filtration. Sequestrins were expressed as genetic fusions to an albumin-binding domain (ABD) and purified by IMAC (**Supplementary Figure S5**). In the biosensor assay, a capture-based setup was employed. An albumin-coated chip surface was used for capture of sequestrins via their albumin-binding domain, after which aSyn (wild type and familial variants) was injected over the surface. The results demonstrated that all four sequestrins bound the familial mutants with affinities comparable to those observed for aSyn wt. Interestingly, analysis of the interaction with the aSyn E46K mutant indicated a somewhat slower dissociation from each of the sequestrins (**Supplementary Figures S6A–D**, **Supplementary Table S2**) compared to the wild type aSyn.

### 3.6 Nuclear magnetic resonance to study the binding epitope on aSyn

**Figure 4:**
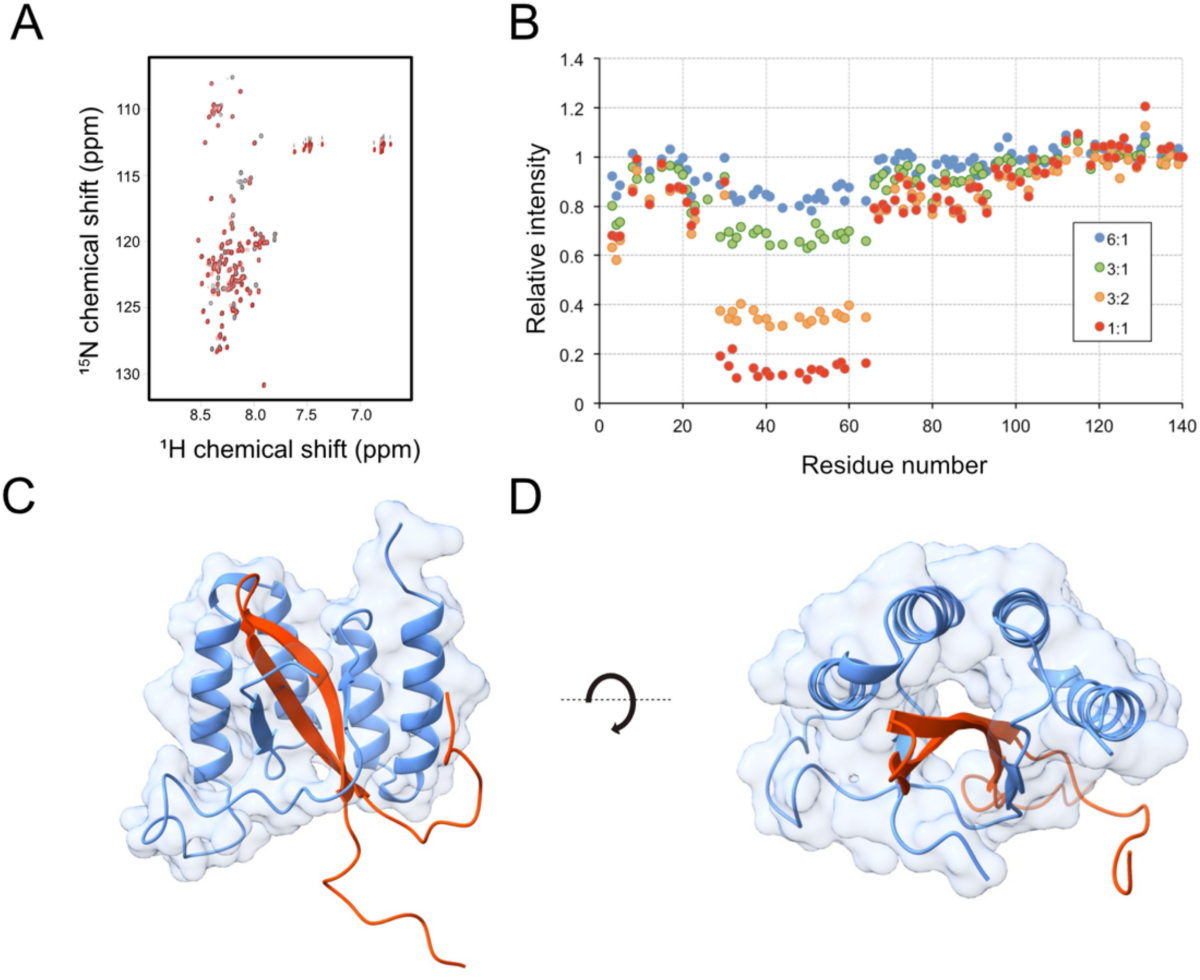
Structure analysis of the aSyn: Sq_aSyn4_ complex by in silico modelling and NMR spectroscopy. **(A, B)** NMR spectroscopy of the Sq_aSyn4_:aSyn_1–140_ complex. **(A)** ^1^H-^15^N HSQC spectrum of uniformly ^15^N-labelled aSyn_1–140_ in absence (black) and presence (red) of 1:1 molar equivalent of Sq_aSyn4_. **(B)** Relative changes in peak intensities along the sequence of aSyn_1–140_ upon addition of Sq_aSyn4_ in molar aSyn: Sq_aSyn4_ ratios from 6:1 to 1:1. **(C, D)** Structure prediction by AlphaFold3, illustrating the interaction of alpha synuclein and Sq_aSyn4_ from two different perspectives. The model is based on a short segment of aSyn comprising the β-hairpin forming N-terminal region (red) and Sq_aSyn4_ (light blue). Confidence metrics for the model were pTM 0.76, ipTM 0.73.

Nuclear magnetic resonance (NMR) spectroscopy was used to investigate which region of aSyn_1–140_ that is recognized by the sequestrins. Sq_aSyn4_ was used as a model molecule for these experiments. Uniformly ^15^N-labeled aSyn_1–140_ was titrated with the sequestrin and 2D ^1^H-^15^N HSQC spectra were recorded for free aSyn and for 6:1, 3:1, 3:2 and 1:1 aSyn: Sq_aSyn4_ molar ratios (**Figure 4A**). Obvious alterations in the HSQC spectrum were observed upon addition of Sq_aSyn4_. The chemical shift changes were small but there were substantial intensity losses for a range of peaks corresponding to amino acid residues 29 to 64 of aSyn (**Figure 4B**), which is the region involved in protein aggregation. At higher Sq_aSyn4_ concentrations, new peaks appeared, indicating slow exchange between free and bound aSyn, which indicates strong binding. These results suggest that partial folding of aSyn into a β-hairpin may be associated with the Sq_aSyn4_:aSyn binding event.

### 3.7 Model of the complex structure

To gain further structural insight into the sequestrin:aSyn interaction, complex models were generated using AlphaFold3 (44). Given the intrinsically disordered nature of full-length aSyn, modeling was focused on the N-terminal epitope identified by NMR (residues 25–65), which encompasses the β-hairpin region (residues 36–57) implicated in aggregation and includes sites of several familial Parkinson’s disease mutations. In agreement with previous studies on sequestrins, the models revealed that aSyn adopts an extended β-strand conformation that is buried within a tunnel-like hydrophobic cavity formed between the two domains of the sequestrin (**Figures 4C, D**, **Supplementary Figure S7**). All four modeled sequestrins, Sq_aSyn2_, Sq_aSyn3_, Sq_aSyn4_, and Sq_aSyn11_, displayed a conserved binding mode, with the sequestrin scaffold maintaining high structural confidence and aSyn exhibiting lower confidence in the flexible terminal regions (**Supplementary Figure S7**). Among the variants, the Sq_aSyn4_:aSyn complex (**Figures 4C, D**, **Supplementary Figure S7C**) showed the highest overall confidence, with the majority of residues in the binding interface reaching per-residue pLDDT scores >90, indicating a well-defined interaction. The model-wide confidence metrics (ipTM = 0.76, pTM = 0.73) further support the reliability of the predicted complex, although some uncertainty in domain orientation may remain. To investigate how familial mutations might affect binding, a model of the Sq_aSyn4_:aSyn complex incorporating the A30P, E46K, and A53T positions was generated (**Supplementary Figure S8**). The mutations are all located within or near the predicted sequestrin-binding region. A30P lies at the N-terminal edge of the binding interface, E46K is situated at the top of the β-hairpin loop engaged by the sequestrin cavity, and A53T is embedded within the core β-strand region that is tightly buried in the complex.

### 3.8 Aggregation inhibition of aSyn by the sequestrins

**Figure 5:**
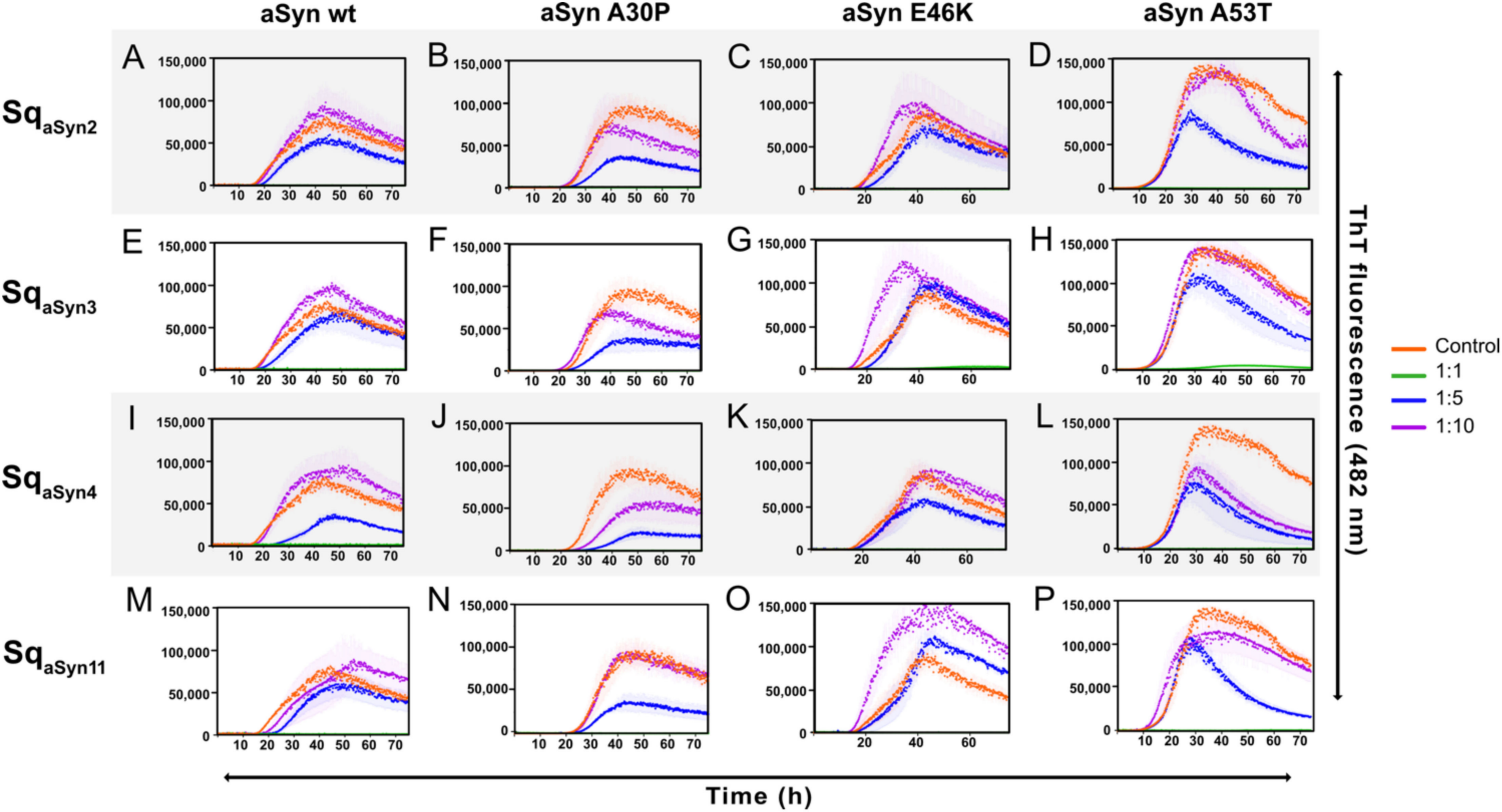
Aggregation time course of aSyn_1–140_ wt or the familial mutants A30P, E46K, and A53T in the absence (orange) or presence of 1:1 (green) or 1:5 (blue) or 1:10 (purple) molar equivalents of the four sequestrins **(A-D)** Sq_aSyn2_, **(E-H)** Sq_aSyn3_, **(I-L)** Sq_aSyn4_, **(M-P)** Sq_aSyn11_. Aggregation was monitored by thioflavin T fluorescence for 72 h, and is presented as mean ± SD.

The four sequestrins, Sq_aSyn2_, Sq_aSyn3_, Sq_aSyn4_, and Sq_aSyn11_, were further evaluated in a thioflavin T (ThT) fluorescence assay to assess their capacity to inhibit aSyn aggregation. Aggregation of aSyn_1–140_ wt and the three familial variants A30P, E46K, and A53T (2, 46) was analyzed with or without the four sequestrins. To ensure correct molar ratios, the proteins were first subjected to size exclusion chromatography. The correct sizes were confirmed by SDS-PAGE (**Supplementary Figure S9**). Each aSyn variant was incubated at a concentration of 70 μM, with the addition of either of the four sequestrins at concentrations of 70 μM (1:1), 14 μM (1:5), 7 μM (1:10), or no sequestrin (0 μM), respectively. ThT was added for monitoring of aSyn aggregation in a plate reader at 37°C for 75 h. The aggregation kinetics of aSyn wt and the three mutants were characterized by a time-dependent increase in fluorescent signal (**Figures 5A–P**, orange). The A53T mutant showed a rapid increase in signal, in agreement with its higher aggregation propensity (47) compared to the other variants (**Figures 5D, H, L, P**, orange). Importantly, co-incubation of aSyn wt or the familial mutants with equimolar concentrations of each sequestrin completely inhibited aggregation (**Figure 5**, green), suggesting that they are potent stochiometric inhibitors of aSyn aggregation. At sub-stoichiometric molar ratios of the sequestrins, more pronounced differences in aggregation-inhibition capacity were observed. At a 1:5 ratio, aggregation of aSyn wt was delayed by all sequestrins (**Figures 5A–P**, blue). Interestingly, Sq_aSyn4_ showed a clear inhibitory effect on the aggregation prone and pathogenic A53T variant at both 1:5 and 1:10 molar ratios (**Figure 5L**, blue and purple), indicating that it is a potent fibrillation inhibitor.

## 4 Discussion

Misfolding and aggregation of the neuronal protein alpha synuclein (aSyn) are central processes driving the pathology of Parkinson’s disease (PD) (1). aSyn has also been observed in other NDDs, including Lewy body dementia and multiple system atrophy. In addition, aSyn has been shown to regulate the fibrillization process of amyloid beta (Aβ) and tau, two proteins involved in Alzheimer’s disease (AD) pathology (5). Aggregates of aSyn are known to trigger the degeneration of affected neurons, leading to dopamine loss and clinical symptoms (5). Preventing aggregation of aSyn or reducing its propagation has been proposed as promising disease-modifying strategies for PD treatment (10).

In this study, we aimed to develop high-affinity sequestrins capable of inhibiting aSyn aggregation by targeting the soluble, monomeric aSyn conformations that predominate in the early stages of the disease. Using phage-display selections from a previously described library (35), we identified several promising sequestrin candidates with K_D_ values in the low nanomolar range (12–30 nM) at physiological temperature, which is comparable to those of monoclonal antibodies targeting aSyn currently in clinical evaluation (13, 14, 16). In line with previous observations on sequestrins binding to other targets (31, 35), CD spectroscopy indicated structural rearrangements in the new Sq:aSyn complexes upon binding. Structural rearrangements in the formed complex were further supported by AlphaFold3 structure predictions, suggesting a β-hairpin structure of aSyn in the complex as well as shielding of the aggregation-prone region of aSyn in a cavity of the sequestrin.

Aggregation of aSyn is driven primarily by either the β-hairpin region (amino acid 36–57) or the hydrophobic NAC domain (amino acid 61–91) (7, 8). To assess which residues that are recognized by the sequestrins, we used NMR spectroscopy, focusing on Sq_aSyn4_ as a representative candidate. Interestingly, residues 29–64 in the N-terminal part of aSyn, which are critical for amyloidogenic misfolding and aggregation, were found to also be involved in binding of the sequestrins. This N-terminal region of aSyn contains several of the disease-related familial mutations, including A30P, E46K, and A53T. We therefore set out to assess the capacity of the sequestrins to inhibit aggregation of both aSyn wt and the familial mutants in an *in vitro* ThT assay. At equimolar concentrations, all sequestrins inhibited aggregation of aSyn wt, and several candidates demonstrated inhibition of aggregation of the familial variants at lower molar ratios. Sq_aSyn4_, in particular, showed an interesting aggregation-inhibition profile also with the more aggregation prone and pathological A53T mutant. To gain further insight into the interaction mechanism, we used AlphaFold3 to model the complexes between aSyn and each of the four sequestrins. The structural predictions showed consistent β-hairpin docking of the aSyn peptide into a central groove formed by the two domains of the sequestrin, shielding key aggregation motifs from solvent exposure (**Supplementary Figure S7**). The highest model confidence was obtained for the Sq_aSyn4_:aSyn complex, which displayed a well-packed core and pLDDT scores above 90 for most interacting residues. As expected, the flexible N- and C-terminal tails of aSyn remained poorly defined in the models. These models also showed that A30P, E46K, and A53T are all located within or adjacent to the predicted binding interface (**Supplementary Figure S8**). A30P maps to the N-terminal boundary of the interaction region, while E46K is situated at the top of the β-hairpin loop engaged by the sequestrin, and A53T lies within the β-strand core deeply buried in the complex. The close proximity of these mutations to key contact residues provides a plausible explanation for the observed differences between sequestrins in their capacity to inhibit aggregation of mutant versus wild-type aSyn.

Immunotherapy-based strategies for treatment of neurogenerative disorders have made significant advances over the years. Currently, nearly a dozen monoclonal antibodies targeting aSyn are in clinical trials for treatment of PD (11, 19). These antibodies, which target different conformations of aSyn, have yielded modest but encouraging effects on disease progression (11, 19). However, non-IgG-based affinity proteins, often referred to as alternative scaffolds, have emerged as promising options for addressing limitations associated with mAbs, such as their large size, complex production, and restricted tissue penetration (23). Among these, affibody molecules have been extensively investigated for various medical applications (23, 48). Building on the affibody platform, the development of the sequestrin scaffold marks a significant advancement. This novel affinity protein design is tailored for interactions with intrinsically disordered proteins and peptides, and has been suggested as an compelling alternative to monoclonal antibodies for prevention of AD and other neurodegenerative diseases (33, 49). Given that such diseases will likely require life-long preventive treatments, sequestrins might offer some advantages. Their small size (~11.2 kDa) enables higher molar dosing compared to antibodies and allows for alternative administration routes, such as subcutaneous injections. Another important factor to enable such long treatments include production costs, to which sequestrins provide an attractive alternative to monoclonal antibodies, as they can be readily produced in bacteria (48). Moreover, unlike antibodies, sequestrins lack immune effector functions, which minimizes the risk of severe side effects.

However, while the small size of sequestrins offers potential advantages over monoclonal antibodies, including higher molar dosing and alternative administration routes, brain uptake remains a key limitation. Previous studies with the sequestrin Z_SYM73_, targeting amyloid-β, demonstrated limited CNS exposure, comparable to that of antibodies (50). To improve brain delivery, additional engineering, such as fusion to a transferrin receptor-binding domain to enable receptor-mediated transcytosis, may be necessary. Furthermore, although sequestrins lack Fc-mediated effector functions, the risk of immunogenicity cannot be excluded and should be carefully assessed. Future studies in relevant *in vivo* models will be essential to evaluate both safety and therapeutic potential.

We used bacterially expressed aSyn to ensure a homogeneous and well-defined starting point for *in vitro* binding studies. While this form lacks post-translational modifications (PTMs), our NMR analysis identified sequestrin binding to residues 29–64, a region where PTMs have been shown to influence aSyn aggregation. Thus, certain PTMs could potentially affect sequestrin binding and activity. To advance the therapeutic development of sequestrins, *in vivo* evaluation in relevant transgenic models will be essential. Such studies are needed to determine whether higher-affinity variants translate into improved neurological outcomes and to assess potential off-target or toxic effects associated with long-term administration.

In summary, the findings of this study underscore the potential of sequestrins as inhibitors of aSyn aggregation and could hopefully pave the way for future investigations in cell and animal models. These results hold promise for advancing sequestrin-based strategies as novel therapeutic approaches for synucleinopathies.

## Data Availability

The original contributions presented in the study are included in the article/**Supplementary Material**. Further inquiries can be directed to the corresponding author.
