# Supplementary material for: Engineered sequestrins inhibit aggregation of pathogenic alpha-synuclein mutants
Source: Front Immunol. 2025 May 16;16:1574755. doi: 10.3389/fimmu.2025.1574755 (PMC12122515; doi:10.3389/fimmu.2025.1574755)
Supplement: Supplementary file 1 [file SupplementaryFile1.pdf]

Supplementary Material

Engineered sequestrins inhibit aggregation of pathogenic alpha-synuclein mutants

Linnea Charlotta Hjelm[1], Wojciech Paslawski[2], Christofer Lendel[3], Siri Flemming Svedmark[1], Per Svenningsson[2], Stefan Ståhl[1], Hanna Lindberg[1], and John Löfblom[1]*

[1]Department of Protein Science, School of Engineering Sciences in Chemistry, Biotechnology and Health, KTH Royal Institute of Technology, Stockholm, Sweden.

[2]Department of Clinical Neuroscience, Karolinska Institutet, 171 76 Stockholm, Sweden.

[3]Department of Chemistry, School of Engineering Sciences in Chemistry, Biotechnology and Health, KTH Royal Institute of Technology, 100 44 Stockholm, Sweden.

***Correspondence:**

John Löfblom: lofblom@kth.se

1 Supplementary Figures and Tables

1.1 Supplementary Tables

Table S1. Kinetic rate constants (k_a and k_d) and equilibrium dissociation constant (K_D) for the interaction of sequestrins and $aSyn_{1\text{-}140}$ at 37°C, as determined by 8K SPR screening. Values for k_a, k_d and K_D were obtained from a 1:1 Langmuir curve fit. Data presented as mean of duplicate injections.

Candidate	k_a [1/Ms]	k_d [1/s]	K_D [nM]
Sq_{aSyn2}	7.46×10^3	1.32×10^{-4}	17.7
Sq_{aSyn3}	3.13×10^3	9.04×10^{-5}	28.9
Sq_{aSyn4}	1.49×10^4	1.88×10^{-4}	12.6
Sq_{aSyn11}	7.70×10^3	2.02×10^{-4}	26.2

Table S2. Kinetic dissociation rate constants (k_d) for sequestrins targeting aSyn wt and familial mutants A30P, E46K, and A53T, as determined by SPR at 25°C. Kinetic dissociation rate constants were estimated using a 1:1 Langmuir model fit.

**Candidate**	**aSyn wt, k_d [1/s]**	**aSyn A30P, k_d [1/s]**	**aSyn E46K, k_d [1/s]**	**aSyn A53T, k_d [1/s]**
Sq_{aSyn2}-ABD	$(1.41 \pm 0.05) \times 10^{-4}$	$(1.52 \pm 0.08) \times 10^{-4}$	$(9.53 \pm 1.31) \times 10^{-5}$	$(1.50 \pm 0.10) \times 10^{-4}$
Sq_{aSyn3}-ABD	$(1.44 \pm 0.00) \times 10^{-4}$	$(1.53 \pm 0.01) \times 10^{-4}$	$(1.16 \pm 0.57) \times 10^{-4}$	$(1.68 \pm 0.00) \times 10^{-4}$
Sq_{aSyn4}-ABD	$(1.16 \pm 0.04) \times 10^{-4}$	$(1.1 \pm 0.05) \times 10^{-4}$	$(9.24 \pm 0.90) \times 10^{-5}$	$(1.27 \pm 0.07) \times 10^{-4}$
Sq_{aSyn11}-ABD	$(1.92 \pm 0.08) \times 10^{-4}$	$(1.99 \pm 0.00) \times 10^{-4}$	$(1.53 \pm 0.10) \times 10^{-4}$	$(2.01 \pm 0.13) \times 10^{-4}$

1.2 Supplementary Figures

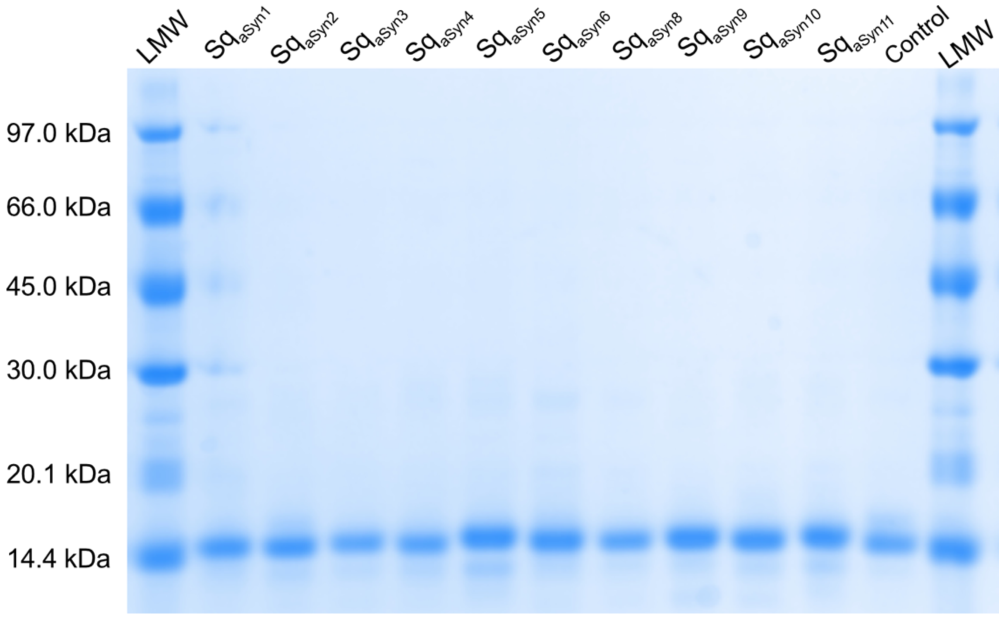

Figure S1. SDS-PAGE analysis of the ten purified Sq_{aSyn}-His_6 proteins (~13 kDa). From left to right: LMW ladder with molecular weighs indicated in kDa, Sq_{aSyn1}, Sq_{aSyn2}, Sq_{aSyn3}, Sq_{aSyn1} Sq_{aSyn4}, Sq_{aSyn5}, Sq_{aSyn6}, Sq_{aSyn8}, Sq_{aSyn9}, Sq_{aSyn10}, Sq_{aSyn11}, control protein, LMW.

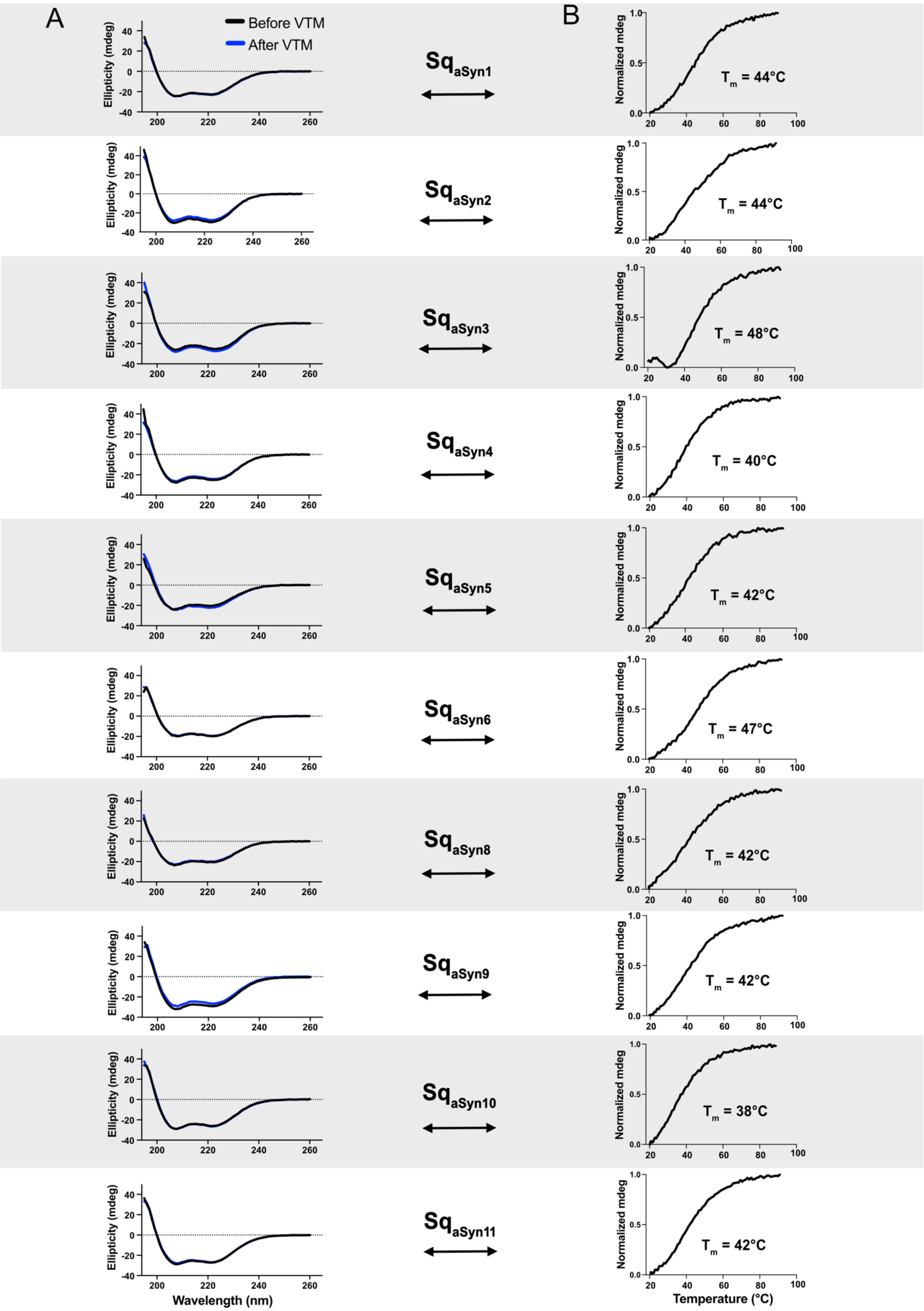
A
B
Before VTM
After VTM
Ellipticity (mdeg)
Wavelength (nm)
Normalized mdeg
Temperature (°C)
Sq aSyn1
T m = 44°C
Sq aSyn2
T m = 44°C
Sq aSyn3
T m = 48°C
Sq aSyn4
T m = 40°C
Sq aSyn5
T m = 42°C
Sq aSyn6
T m = 47°C
Sq aSyn8
T m = 42°C
Sq aSyn9
T m = 42°C
Sq aSyn10
T m = 38°C
Sq aSyn11
T m = 42°C

Figure S2. Circular dichroism (CD) spectroscopy of the ten phage-selected aSyn-binding sequestrins, including secondary structure determination and variable temperature measurements (VTM). (A) Secondary structure determination before (black) and after (blue) VTM. (B) VTM between 20 – 90°C at 221 nm and while heating 1°C/min. Melting temperature for each sequestrin is indicated in the figure.

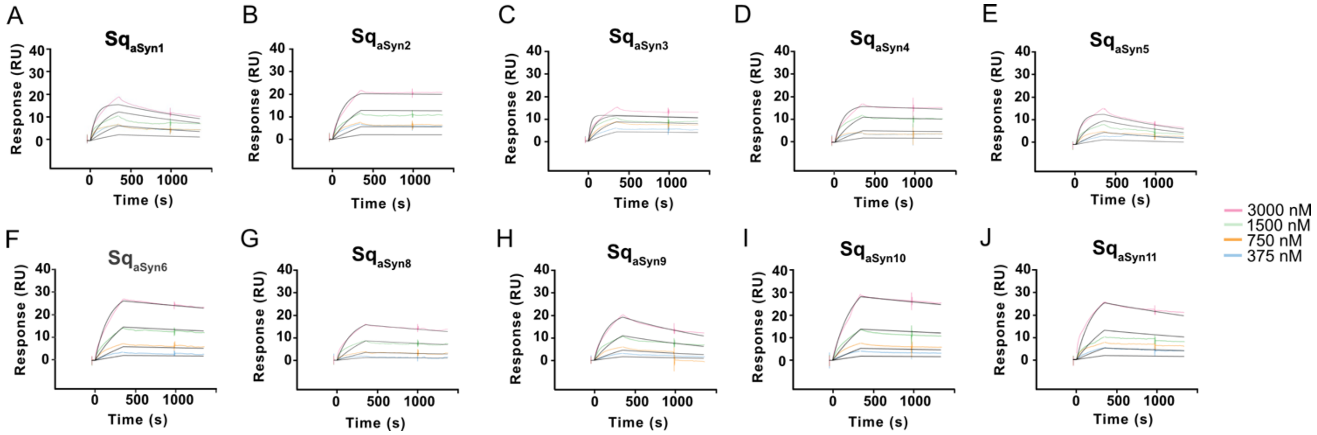

Figure S3. Surface plasmon resonance (SPR)-based affinity screening of the ten aSyn-binding sequestrins and $aSyn_{1-140}$ at 25°C. Sequestrins (A) Sq_{aSyn1}, (B) Sq_{aSyn2}, (C) Sq_{aSyn3}, (D) Sq_{aSyn4}, (E) Sq_{aSyn5}, (F) Sq_{aSyn6}, (G) Sq_{aSyn8}, (H) Sq_{aSyn9}, (I) Sq_{aSyn10}, (J) Sq_{aSyn11} were injected in duplicates over an aSyn-coated sensor chip surface and in dilution series spanning 3000 nM to 375 nM. Colored curves are referenced raw data, and black lines are fitted curves.

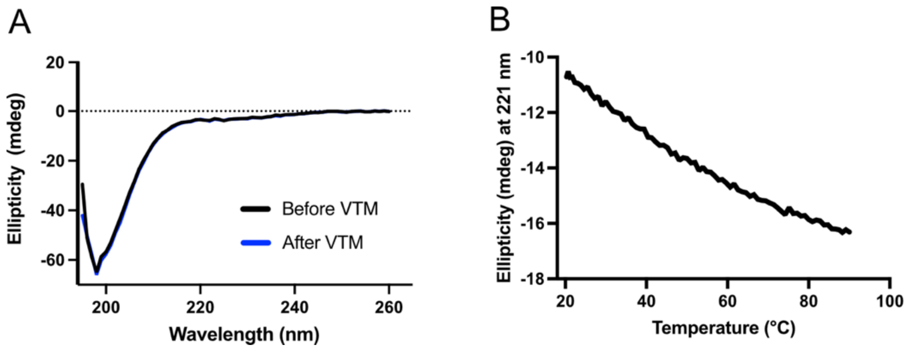

Figure S4. Circular dichroism (CD) spectroscopy of secondary structure and variable temperature measurement (VTM) of aSyn$_{1\text{-}140}$. (A) Secondary structure determination at 20°C and between 195-260 nm of free aSyn$_{1\text{-}140}$ before (black) and after (blue) thermal melting. (B) VTM of aSyn$_{1\text{-}140}$ between 20-95°C at 221 nm.

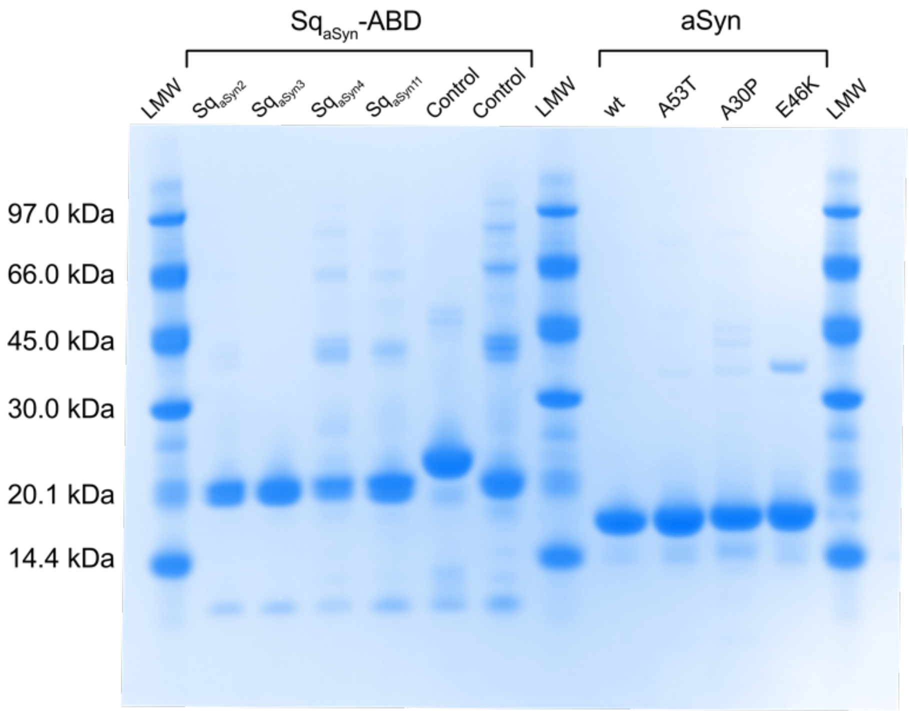

Figure S5. SDS-PAGE analysis of the four purified Sq$_{aSyn}$-ABD candidates (~20 kDa) and four aSyn variants, including wild-type, A53T, A30P, E46K (~15 kDa). From left to right: LMW ladder with molecular weighs indicated in kDa, Sq$_{aSyn2}$-ABD, Sq$_{aSyn3}$-ABD, Sq$_{aSyn4}$-ABD, Sq$_{aSyn11}$-ABD, control protein, control protein, LMW, aSyn-wt, aSyn-A53T, aSyn-A30P, aSyn-E46K, LMW.

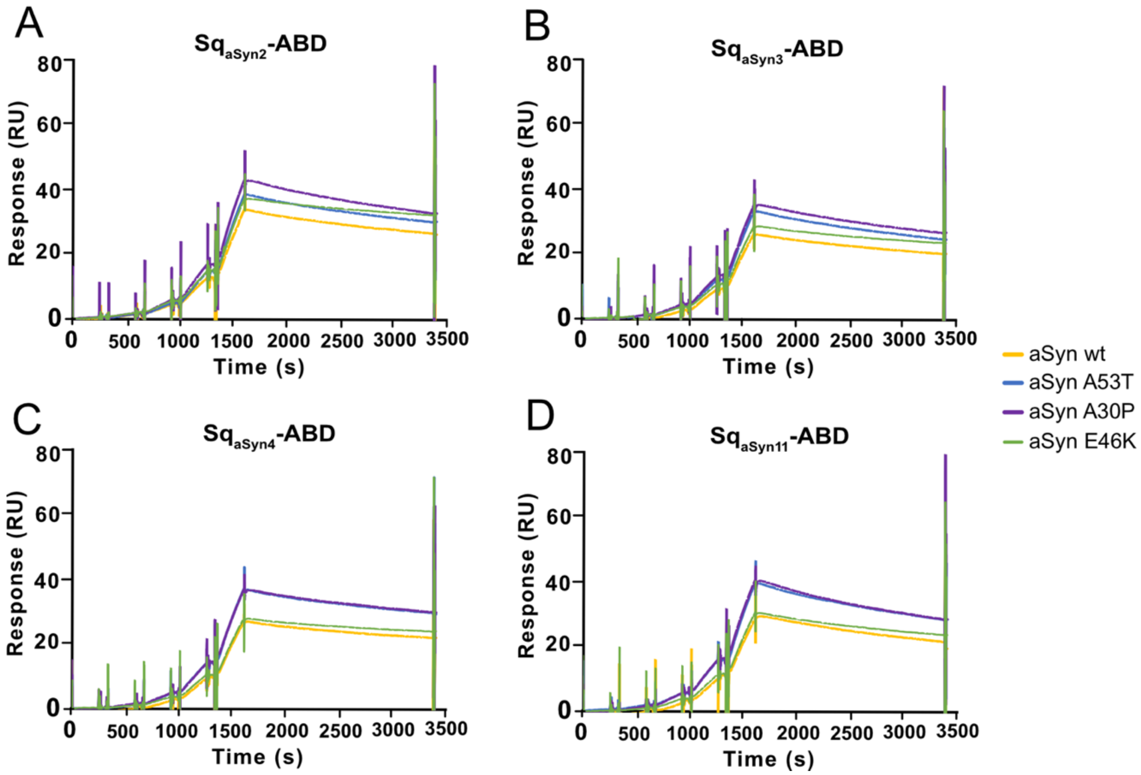

Figure S6. Surface plasmon resonance (SPR) sensorgrams showing the interaction between four sequestrin-ABD format constructs and aSyn wt and three familial mutants. (A) Sq_{aSyn2}-ABD, (B) Sq_{aSyn3}-ABD, (C) Sq_{aSyn4}-ABD, and (D) Sq_{aSyn11}-ABD were captured on an HSA coated biosensor chip, followed by injection of the different aSyn proteins, as indicated in the figure (wt: yellow, A53T: blue, A30P: purple, E46K: green). Proteins were injected in a single cycle at five concentrations (24000, 8000, 2667, 888, 296 nM) and dissociation was analyzed for 1,800 s. Kinetic dissociation rate constants were estimated using a 1:1 Langmuir model fit.

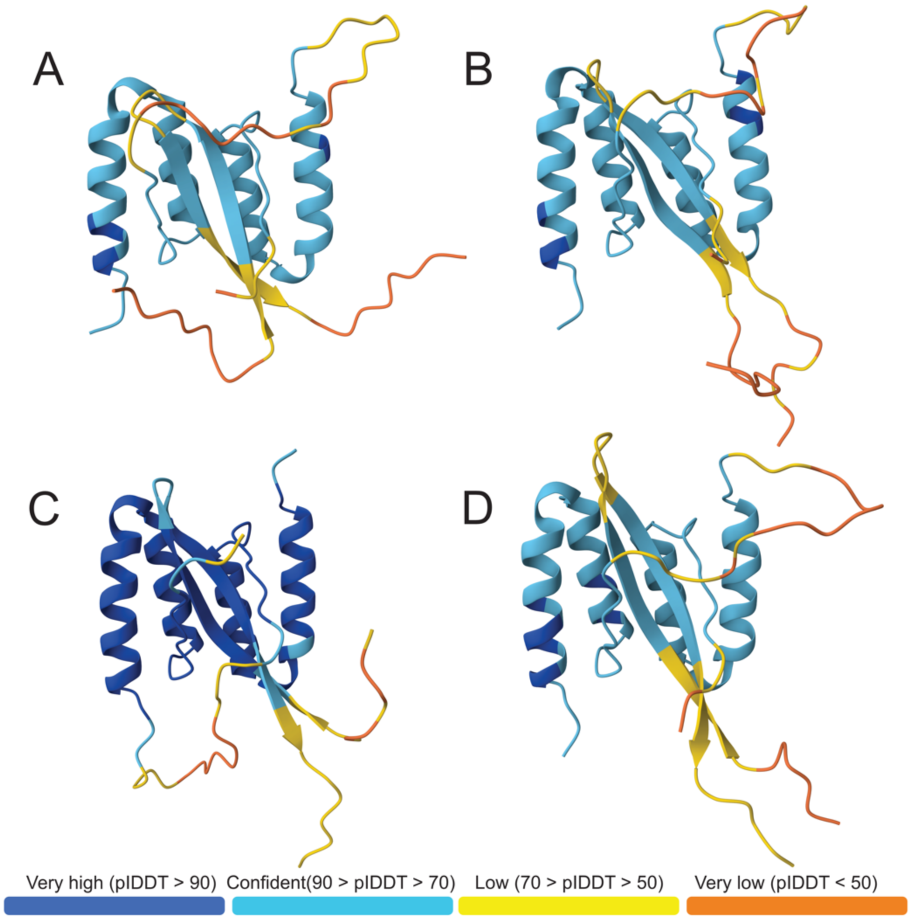

Figure S7. Structure predictions by AlphaFold3 of alpha-synuclein bound to sequestrins.
Predicted complex structures showing the interaction between alpha-synuclein (residues 25–65) and four different sequestrin variants: (A) Sq_{aSyn2}, (B) Sq_{aSyn3}, (C) Sq_{aSyn4}, and (D) Sq_{aSyn11}. The color gradient represents per-residue confidence (pLDDT scores) from AlphaFold3, with dark blue indicating very high confidence (pLDDT > 90), light blue indicating confident regions (90 > pLDDT > 70), yellow indicating low confidence (70 > pLDDT > 50), and orange/red indicating very low confidence (pLDDT < 50).

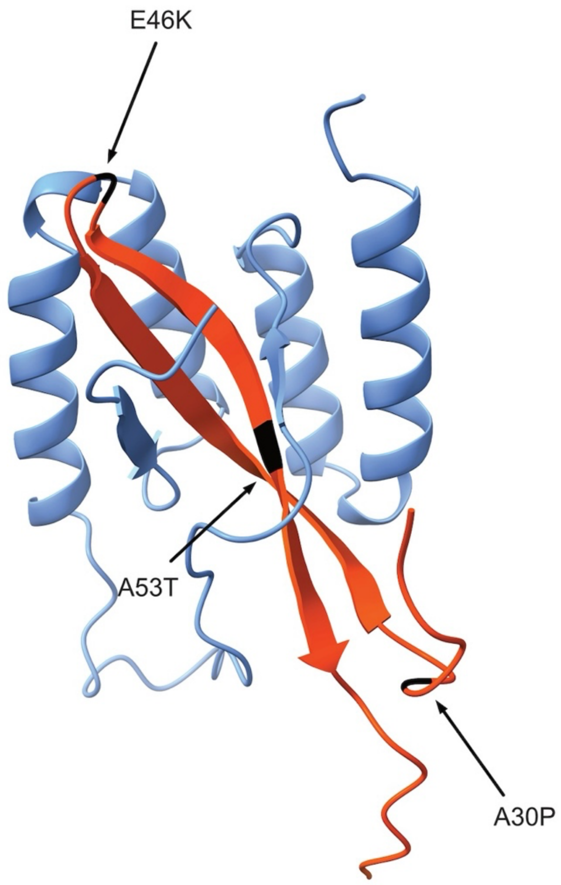

Figure S8. Structure prediction by AlphaFold3 of alpha-synuclein in complex with sequestrin Sq_{aSyn4}, highlighting familial Parkinson's disease mutations. The predicted complex structure shows the interaction between Sq_{aSyn4} (blue) and the N-terminal region of alpha-synuclein (red), modeled using AlphaFold3. Residues corresponding to three familial Parkinson's disease mutations, A30P, E46K, and A53T, are indicated with arrows.

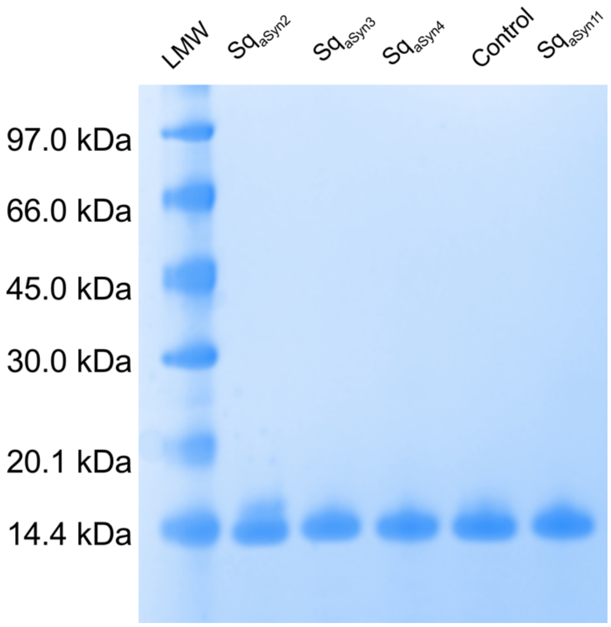

Figure S9. SDS-PAGE analysis of four size exclusion chromatography purified aSyn-binding sequestrins (~13 kDa). From left to right: LMW ladder with molecular weighs indicated in kDa, Sq_{aSyn2}-His_6, Sq_{aSyn3}-His_6, Sq_{aSyn4}-His_6, control protein, Sq_{aSyn11}-His_6.
